# Clinical, Radiographic and Fusion Comparison of Oblique Lumbar Interbody Fusion (OLIF) stand-alone and OLIF with posterior pedicle screw fixation in patients with degenerative spondylolisthesis

**DOI:** 10.1186/s12891-023-06985-8

**Published:** 2023-10-30

**Authors:** Wenhao Zhao, Chuanli Zhou, Hao Zhang, Jianwei Guo, Jialuo Han, Antao Lin, Yan Wang, Xuexiao Ma

**Affiliations:** Department of Spine Surgery, The Affiliated Hospital of Qingdao University, 59 Haier Road, Qingdao, Shandong 266000 China

**Keywords:** Spinal fusion, Spinal stenosis, Oblique lumbar interbody fusion, Spondylolisthesis, Percutaneous pedicle screw fixation

## Abstract

**Purpose:**

To compare the outcomes and characteristics of oblique lumbar interbody fusion stand-alone (OLIF-SA) and OLIF with posterior pedicle screw fixation (OLIF-PPS) in the treatment of Grade I or Grade II degenerative lumbar spondylolisthesis.

**Patients and methods:**

Between January 2019 and May 2022, 139 patients with degenerative spondylolisthesis were treated with OLIF-SA (n = 85) or OLIF-PPS (n = 54). The clinical and radiographic records were reviewed.

**Results:**

The clinical and radiographic outcomes were similar in both groups. The operative time and intraoperative blood loss in the OLIF-SA group were lower than those in the OLIF-PPS group (P < 0.05). However, the OLIF-PPS group had significantly better disc height (DH) and postoperative forward spondylolisthesis distance (FSD) improvement at 6 months (P < 0.05). The OLIF-PPS group had a significantly lower cage subsidence value than the OLIF-SA group (P < 0.05). Improvement of the lumbar lordotic angle (LA) and fusion segmental lordotic angle (FSA) in the OLIF-PPS group was significantly better than that in the OLIF-SA group (P < 0.05). In terms of fusion types, the OLIF-SA group tended to undergo fusion from the edge of the vertebral body. Fusion in the OLIF-PPS group began more often in the bone graft area of the central cage of the vertebral body. The fusion speed of the OLIF-SA group was faster than that of the OLIF-PPS group.

**Conclusion:**

OLIF-SA has the advantages of a short operative time, less intraoperative blood loss, and reduced financial burden, while PPS has incomparable advantages in the reduction of spondylolisthesis, restoration of lumbar physiological curvature, and long-term maintenance of intervertebral DH. In addition, the SA group had a unique vertebral edge fusion method and faster fusion speed.

## Introduction

Degenerative lumbar spondylolisthesis is the most common type of lumbar spondylolisthesis, mainly occurring in elderly individuals and often leading to lower back pain and nerve root pain. A variety of lumbar fusion surgeries have developed for the treatment of lumbar spondylolisthesis [1–3].

Oblique lumbar interbody fusion (OLIF) is an anterior minimally invasive lumbar fusion method that can increase the height of the intervertebral disc through the use of a cage to reduce the bulging of the intervertebral disc and extend the hypertrophic ligamentum flavum to achieve indirect decompression. The advantages of OLIF include avoiding damage to the posterior paravertebral soft tissue, maintaining the stability of the posterior column, and causing less nerve stimulation during the operation, which have attracted much attention in clinical practice [4–7]. However, the rate of cage subsidence after OLIF stand-alone (OLIF-SA) surgery is approximately 30%, which can affect orthopedic surgery and even lead to decompression failure [5, 8, 9]. Our clinical experience and some reports suggest that posterior percutaneous pedicle screw placement can reduce the incidence of cage subsidence. However, He et al. suggested that OLIF-SA and OLIF with posterior pedicle screw fixation (OLIF-PPS) have similar clinical and radiological results and a similar incidence of complications [10]. Guo et al. reported that the OLIF Pivox technique had the same clinical results, imaging findings, and incidence of complications as OLIF-PPS [11]. However, Ge et al.‘s report suggested that additional lateral plate fixation during OLIF surgery had no effect on preventing cage subsidence [12].

The current literature generally has limitations, such as small sample size, inconsistent disease types, random research objectives, and short follow-up time, and some study conclusions are not consistent [10–16]. The biggest limitation is that currently, all studies related to lumbar fusion performed only a simple statistical analysis of the fusion rate, and we found in our clinical work that the fusion performance of OLIF-SA and OLIF-PPS is not the same. To our knowledge, this study has the largest sample size and the longest average follow-up of OLIF-SA and OLIF-PPS in the treatment of degenerative lumbar spondylolisthesis. We analyzed the differences between the two methods from the three aspects of clinical results, imaging findings and fusion performance to provide a reference.

## Materials and methods

### Subjects

Consent was obtained from each patient and the study was approved by ethics committee of Affiliated Hospital of Qingdao University (approval number: QYFY WZLL 28,213). We searched the medical records of patients with degenerative lumbar spondylolisthesis who underwent OLIF surgery in our hospital from January 2019 to May 2022. Finally, a total of 139 patients were included in the retrospective study, including 85 in the OLIF-SA group and 54 in the OLIF-PPS group. The inclusion criteria were as follows: (1) patients were diagnosed with degenerative lumbar spondylolisthesis, Meyerding Grade II or less, and conservative treatment for more than 3 months was ineffective; (2) patients underwent OLIF-SA or OLIF-PPS; and (3) ≤ 2 surgical segments were involved. The exclusion criteria were as follows: (1) severe spinal stenosis; (2) severe lumbar spondylolisthesis (> Meyerding Grade II); (3) severe osteoporosis; (4) morbid obesity (body mass index (BMI) > 35 kg/m2); (5) history of lumbar surgery, infection, tumor or other serious diseases; and (6) preoperative segmental spontaneous fusion of the intervertebral space.

### Surgical Technique

After administration of general anesthesia, the patient was placed in the right decubitus position. The right arm was straightened to the front and placed on the pallet. The left arm was straightened to the front with the elbow joint flexed 90° and cushioned with pads. The patient’s back was placed perpendicular to the plane of the operating table, and the waist was padded with an arc cushion to make the surgical segment parallel to the horizontal plane. The patient’s right leg was straightened, and the left leg was placed in the hip flexion and knee flexion positions to relax the psoas major muscle. After the body was positioned, the patient was fixed with medical tape at the shoulder level and hip level, and both legs were fixed with medical straps. Then, a C-arm was used to locate and mark the surface projection of the intervertebral space of the surgical segment. An oblique incision was made 4–6 cm from the midpoint of the intervertebral space. The skin and subcutaneous tissue were cut in turn, and the external oblique muscle, internal oblique muscle, transverse abdominal muscle and transverse abdominal fascia were bluntly dissected along the direction of the muscle fibers to provide an entrance to the retroperitoneal space. The peritoneum and its contents were pulled ventrally, and the psoas major muscle was pulled dorsally to expose the surface of the vertebral body.

**Fig. 1:**
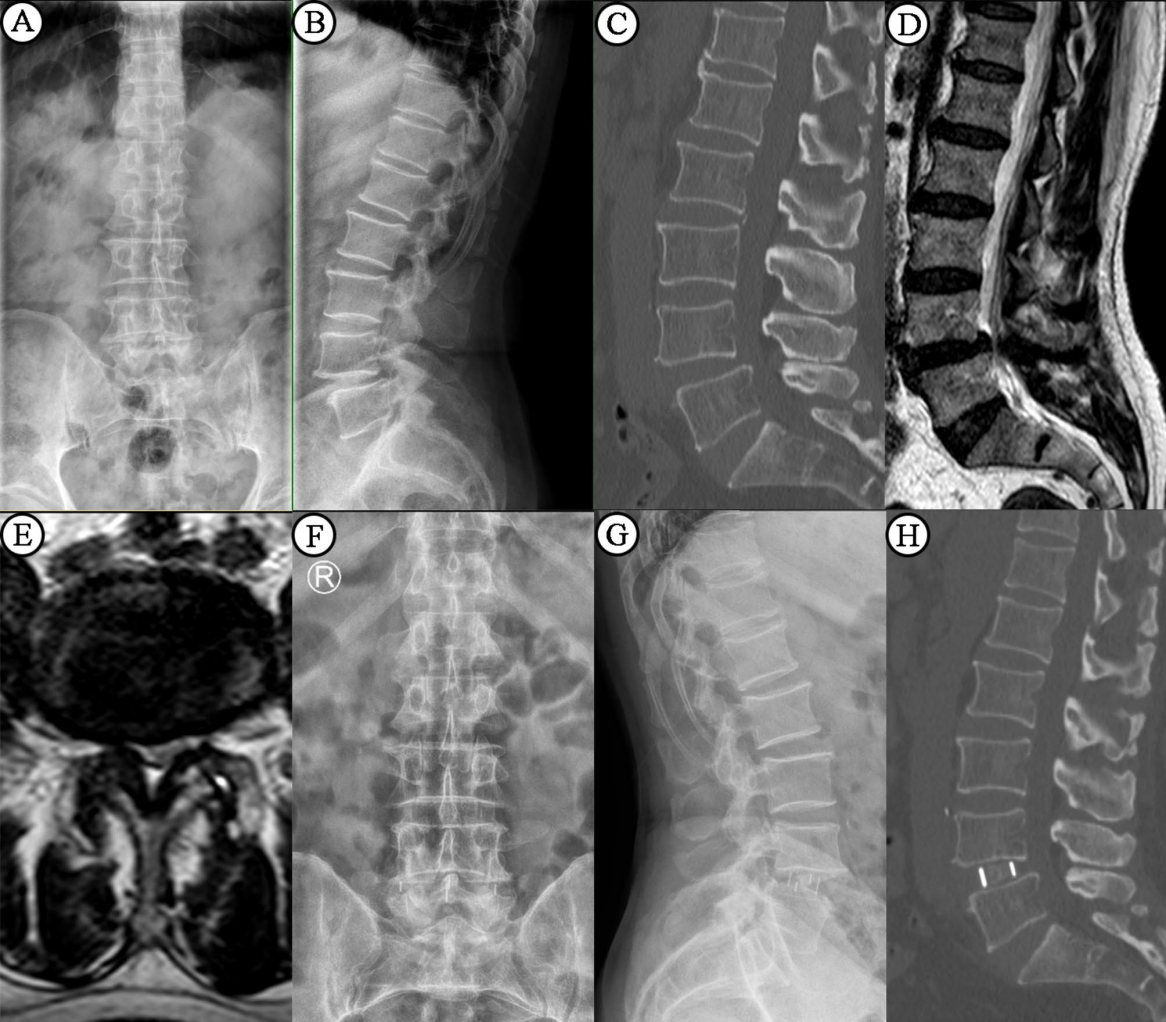
A 64-year-old woman in the SA group was diagnosed with L4-5 lumbar spondylolisthesis. Preoperative x-rays and computed tomography scans showed Grade I spondylolisthesis (**A**-**C**). Magnetic resonance imaging images showed spinal stenosis and nerve root canal stenosis (**D**-**E**). The patient underwent L4-5 OLIF stand-alone (**F**-**H**).

**Fig. 2:**
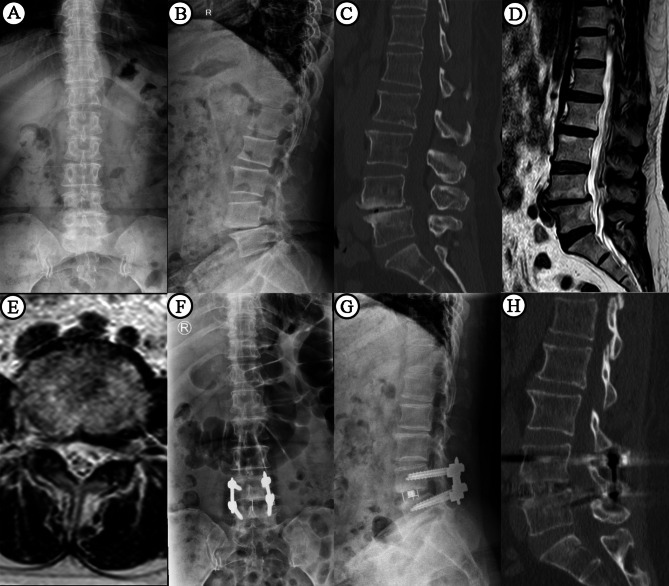
A 54-year-old woman in the PPS group was diagnosed with L4-5 lumbar spondylolisthesis. Preoperative x-rays and computed tomography scans showed Grade I spondylolisthesis (**A**-**C**). Preoperative magnetic resonance imaging images showed spinal stenosis (**D**-**E**). The patient underwent L4-5 OLIF with pedicle screw placement (**F**-**G**). Sagittal computed tomography images at 12 months postoperatively showed that the interbody fusion was achieved in the central region of the vertebral body (**H**)

A Kirschner wire was fixed in the intervertebral space, and the target segment was located and confirmed by C-arm fluoroscopy. The depth of the incision was measured along the Kirschner wire with a sounder, and the appropriate expansion channel was selected. The position and angle of the expansion channel were adjusted, and the channel light source was installed. The annulus fibrosus was cut with a long-handled sharp knife, the nucleus pulposus tissue was removed with nucleus pulposus forceps, and the endplate was treated with various types of reamers and endplate scrapers. Finally, the contralateral annulus fibrosus was cut. Test molds were inserted into the intervertebral space from small to large sizes to determine the model of the cage. The artificial bone presoaked in normal saline was put into the cage. The cage was inserted into the intervertebral space, first obliquely and then vertically, and completely placed. The appropriate position of the cage was confirmed by C-arm fluorescence microscopy. The operation area was rinsed with a large amount of normal saline, and the wound was sutured layer by layer after complete hemostasis (Fig. 1). Patients also undergoing posterior pedicle screw fixation were moved into the conventional prone position. All patients underwent Mazor Renaissance robot-assisted percutaneous pedicle screw placement (Fig. 2). All patients were required to exercise on the second or third day after surgery (depending on the patient’s tolerance and physical condition). All patients were required to avoid violent activities and wear thoracolumbar braces for 3 months after surgery. Patients usually did not use analgesics after exercise.

### Clinical Indicators

The clinical information collected included age, sex, BMI, operative segment, operative time, intraoperative blood loss, complications and length of hospital stay. The visual analog scale (VAS) and Oswestry disability index (ODI) were completed before the operation, one week after the operation, three months after the operation and one year after the operation. Computed tomography (CT) was used to evaluate the fusion and endplates at 6 months and one year after the operation.

### Radiographic Parameter Measurement

**Fig. 3:**
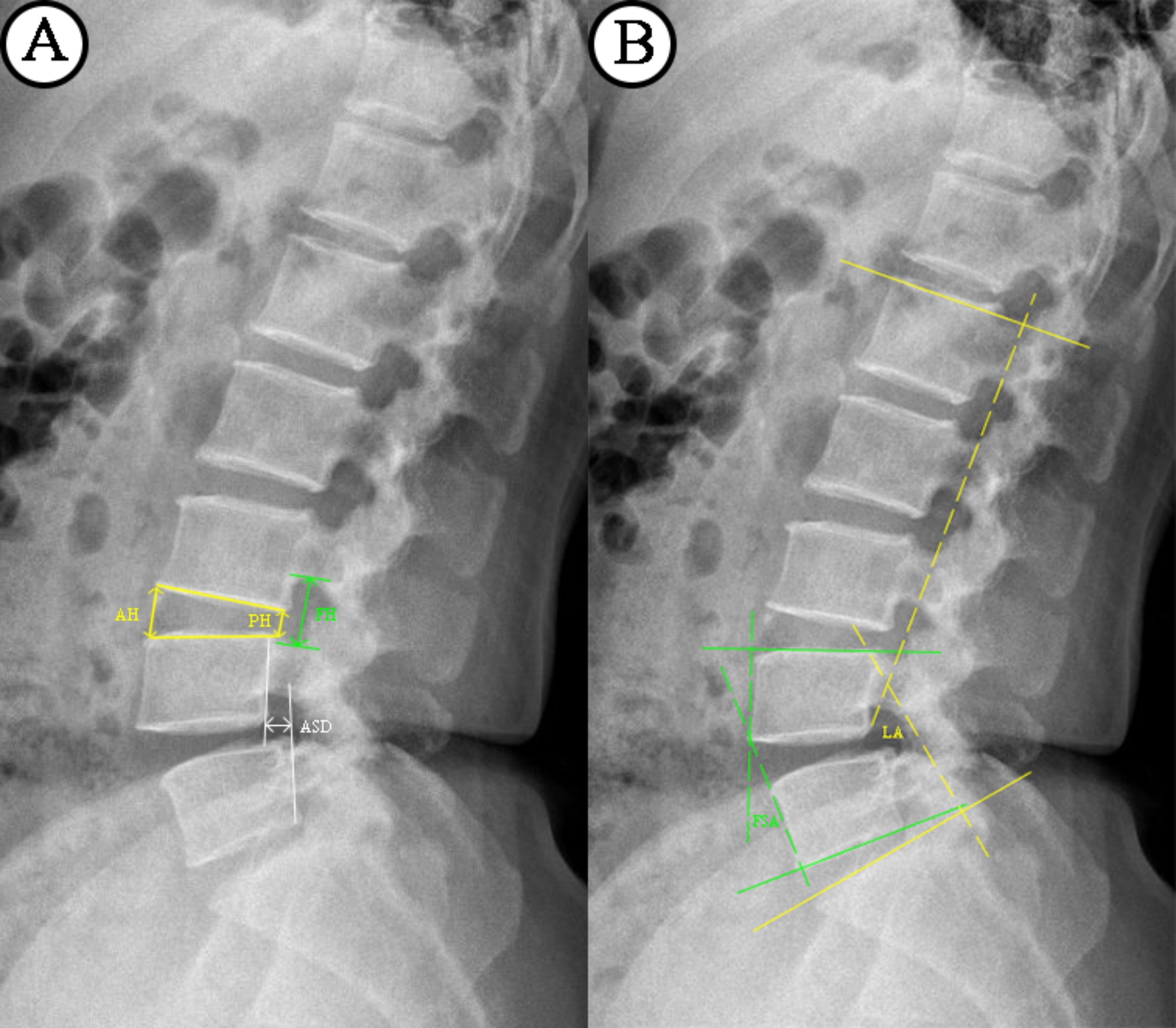
Length parameters measured on lateral X-rays (**A**). Disc height= (AH + PH)/2; foraminal height = FH; Anterior spondylolisthesis distance = ASD. Angle parameters measured on lateral X-rays (**B**). Segmental lordotic angle = FSA; lumbar lordotic angle = LA.

X-ray examination was performed before the operation, after the operation and at each follow-up. CT was performed before the operation, 6 months after the operation and 1 year after the operation. Magnetic resonance imaging was performed before and after the operation and when cage subsidence was found during the follow-up. As shown in Fig. 2, the disc height (DH), anterior spondylolisthesis distance (ASD), foraminal height (FH), fusion segmental lordotic angle (FSA), and lumbar lordotic angle (LA) were evaluated on the X-ray images. DH was measured as the average anterior and posterior DH on the lateral X-ray image. ASD was measured as the relative distance between the posterior cortices of the two vertebral bodies. FH was the vertical distance between the horizontal tangents of the lower edge of the upper vertebral pedicle and the upper edge of the lower vertebral pedicle, which was measured on lumbar CT sagittal images. The FSA was the angle between the lower endplate of the upper vertebral body and the upper endplate of the lower vertebral body. The LA was the angle between the upper endplate of L1 and the upper endplate of S1. The fusion condition was evaluated by CT (Fig. 3). This measurement was carried out by two experienced physicians respectively, and the classification of fusion types was evaluated on the three-dimensional CT of patients’ postoperative reexamination. Nirmal D. Patil et al. [17]. Proposed a new evaluation method of interbody fusion. In their article, they mentioned CT imaging techniques to be superior to plain film radiographs in determining the presence of bony fusion However, CT imaging did not accurately determine the extension of bony fusion present as confirmed by histologic analysis. Therefore, the fusion grading standard was based on the Bridwell interbody fusion grading system [18], and Grade I and Grade II were considered successful. The classification of interbody fusion has not been supported by relevant literature. We temporarily classified it as association for the study of internal fixation (AO type) and biological osteosynthesis (BO type) according to the principle of fracture healing. Specifically, on CT images, the AO type starts to fuse in the bone graft area of the cage and expands around, while in the BO type, osteophytes form at the edge of the vertebral body or at the periphery of the cage (the periphery is limited to the area that has no obvious correlation with the cage bone graft area), and the first fusion occurs at the periphery of the vertebral body. The degree of settlement was scored with the grading system reported by Marchi et al., in which Grade 0 and Grade I were considered low-grade subsidence and Grade II and Grade III were considered high-grade subsidence [19]. All data were measured by two experienced surgeons independently, and the average value of the two measurements was used for the final analysis.

### Statistical analysis

SPSS 25.0 software for Windows (IBM Co, Armonk, NY, USA) was used for statistical analysis. Descriptive statistics are reported as the mean ± standard deviation, frequency or percentage when appropriate. Continuous data with a normal distribution were analyzed by Student’s t test, and the Mann‒Whitney U test was used for continuous data with a nonnormal distribution. The chi-square test or Fisher’s exact test was used for categorical variables. P < 0.05 was considered statistically significant.

## Results

### Demographic data and perioperative indicators

The demographic and perioperative information of each group of patients is shown in Table 1. A total of 139 patients (47 males and 92 females) participated in the study. The average age of the patients was 62.99 ± 8.27 years (43–84 years). Most of the surgical segments were at the L4/5 level (n = 106, 76.25%). There was no significant difference in age, sex, BMI or length of hospital stay between the two groups (p > 0.05). The operation time and intraoperative blood loss in the OLIF-PPS group were significantly higher than those in the OLIF-SA group (p < 0.05).

**Table 1:** General information and perioperative indicator

	All	SA group	PPS group	P
Number of patients	139	85	54	
Age(year)	62.99 ± 8.27	63.61 ± 8.20	62.00 ± 8.36	0.264
Sex, n (%)				
Men	47	32(37.6%)	15(27.8%)	0.272
Women	92	53(62.4%)	39(72.2%)	
BMI		26.02 ± 2.92	26.49 ± 4.27	0.445
Fusion segment, n (%)				
L2/3	2(1.45%)		2(3.7%)	
L3/4	31(22.30%)	23(27.1%)	8(14.8%)	
L4/5	106(76.25%)	62(72.9%)	44(81.5%)	
Operation time (min)		99.06 ± 38.74	250.09 ± 74.93	< 0.001
Hospital stays (day)		7.98 ± 1.54	7.87 ± 2.71	0.062
Intraoperative blood loss (ml)		26.76 ± 9.90	60.37 ± 21.63	< 0.001

Values are presented as mean ± standard deviation or frequency (percentage)

SA: Oblique lumbar interbody fusion stand alone; PPS: Oblique lumbar interbody fusion with posterior pedicle screw fixation

Statistically significant at p < 0.05

### Radiographic outcomes

There was no significant difference in the DH, change in DH or FH between the two groups immediately after the operation (p > 0.05), but at the 6-month follow-up, the DH of the OLIF-PPS group was significantly greater than that of the OLIF-SA group (13.25 ± 1.76 mm and 11.47 ± 1.85 mm, p < 0.001), and the cage subsidence value of the OLIF-SA group was significantly greater than that of the OLIF-PPS group (1.34 ± 0.88 mm and 0.08 ± 0.10, p < 0.001). In terms of reduction of spondylolisthesis, the reduction improvement in the OLIF-PPS group was also significantly better than that in the OLIF-SA group (4.79 ± 1.60 mm and 3.43 ± 1.37 mm, p < 0.001). The immediate postoperative fusion segmental lordosis in the OLIF-SA group was slightly smaller than that in the OLIF-PPS group, but the difference was not significant (21.22 ± 5.91° and 22.52 ± 5.58, P = 0.20). However, the immediate postoperative improvement in the OLIF-SA group was significantly lower than that in the OLIF-PPS group (3.51 ± 3.81° and 5.33 ± 3.89°, p < 0.05). The immediate postoperative LA (54.2 ± 8.78° and 54.02 ± 61.05°, p < 0.001) and immediate postoperative improvement value (13.30 ± 9.39° and 12.41 ± 59.84°, p < 0.001) of the OLIF-PPS group were significantly higher than those of the OLIF-SA group. More details are shown in Table 2.

**Table 2:** Radiological outcomes

	SA group	PPS group	P
Pre DH	8.43 ± 2.22	8.70 ± 1.80	0.435
Post DH	12.81 ± 1.79	13.33 ± 1.77	0.1
6 Months DH	11.47 ± 1.85	13.25 ± 1.76	< 0.001
Post Improvement	4.38 ± 2.09	4.63 ± 2.07	0.499
6 Months Cage Subsidence Value	1.34 ± 0.88	0.08 ± 0.10	< 0.001
Pre ASD	6.24 ± 1.56	6.71 ± 1.92	0.046
Post ASD	2.81 ± 1.67	1.92 ± 1.07	< 0.001
Post Improvement	3.43 ± 1.37	4.79 ± 1.60	< 0.001
Pre FH	15.95 ± 2.77	15.89 ± 3.25	0.702
Post FH	22.08 ± 3.19	22.09 ± 2.70	0.941
Post Improvement	6.13 ± 3.07	6.20 ± 3.03	0.663
Pre FSA	17.71 ± 5.58	17.19 ± 5.45	0.584
Post FSA	21.22 ± 5.91	22.52 ± 5.58	0.20
Post Improvement	3.51 ± 3.81	5.33 ± 3.89	0.002
Pre LA	41.62 ± 12.19	45.72 ± 12.01	0.122
Post LA	54.02 ± 61.05	54.2 ± 8.78	< 0.001
Post Improvement	12.41 ± 59.84	13.30 ± 9.39	< 0.001

Values are presented as mean ± standard deviation

SA: Oblique lumbar interbody fusion stand alone; PPS: Oblique lumbar interbody fusion with posterior pedicle screw fixation. DH: disc height. ASD: anterior spondylolisthesis distance; FH: foraminal height; FSA: fusion segmental lordotic angle; LA: lumbar lordotic angle

Statistically significant at p < 0.05

### Fusion outcomes

Seventy-eight (91.76%) patients in the OLIF-SA group achieved fusion at the 6-month follow-up, and all achieved fusion at the 1-year follow-up (n = 85); 46 (85.1%) patients in the OLIF-PPS group achieved fusion at the 6-month follow-up, and all achieved fusion at the 1-year follow-up (n = 54). At the 6-month follow-up, the fusion rate in the OLIF-SA group was higher than that in the OLIF-PPS group, but the difference was not significant. Among all the patients who achieved fusion, 25.88% (22/85) in the OLIF-SA group exhibited the AO type and 74.12% (63/85) exhibited the BO type; 1.85% (1/54) in the OLIF-PPS group exhibited the AO type and 98.15% (53/54) exhibited the BO type. There was a significant difference in the fusion type between the two groups (p < 0.001), More details are shown in Table 3.

**Table 3:** Surgical segment fusion information

	SA group	PPS group	P
6 months			
Grade I	68	37	
Grade II	10	9	
Grade III	7	8	
Grade IV			
Fusion rate	91.76%	85.19%	0.223
12 months			
Grade I	82	48	
Grade II	3	6	
Grade III	/	/	
Grade IV	/	/	
Fusion rate	100%	100%	
Fusion type			< 0.001
AO type	22(25.88%)	1(1.85%)	
BO type	63(74.12%)	53(98.15%)	

Values are presented as frequency (percentage)

SA: Oblique lumbar interbody fusion stand alone; PPS: Oblique lumbar interbody fusion with posterior pedicle screw fixation

Statistically significant at p < 0.05

### Clinical outcomes

The patients in the OLIF-SA group and the OLIF-PPS group showed significant improvement in all functional indicators compared with those before surgery (Table 4). At the follow-up visit 1 week after the operation, the VAS score and ODI of patients in the OLIF-PPS group were significantly higher than those in the OLIF-SA group (4.72 ± 16.5 and 4.08 ± 1.37, p < 0.05), but at the follow-up visits 3 months and 1 year after the operation, the two values in the OLIF-PPS group were lower than those in the OLIF-SA group, but the difference was not significant. There were differences in the VAS score for leg pain between the two groups in each follow-up period, but the differences were not significant.

**Table 4:** Clinical outcomes

	SA group	PPS group	P
Back pain VAS score			
Preoperative	7.06 ± 0.75	7.15 ± 0.66	0.394
Postoperative, 1 week	4.08 ± 1.37	4.72 ± 1.65	0.027
Postoperative, 3 months	2.02 ± 1.31	1.74 ± 0.52	0.899
Postoperative, 1 year	1.82 ± 0.71	1.81 ± 0.93	0.486
Leg pain VAS score			
Preoperative	6.32 ± 1.58	6.09 ± 1.48	0.221
Postoperative, 1 week	3.62 ± 1.59	3.65 ± 1.65	0.895
Postoperative, 3 months	1.94 ± 1.20	1.70 ± 0.54	0.797
Postoperative, 1 year	1.34 ± 0.57	1.39 ± 0.49	0.633
Oswestry disability index score			
Preoperative	30.52 ± 5.36	31.43 ± 4.52	0.546
Postoperative, 1 week	16.35 ± 2.92	17.5 ± 2.94	0.037
Postoperative, 3 months	8.28 ± 2.13	7.78 ± 2.52	0.143
Postoperative, 1 year	5.98 ± 1.90	5.67 ± 1.84	0.29

Values are presented as mean ± standard deviation

SA: Oblique lumbar interbody fusion stand alone; PPS: Oblique lumbar interbody fusion with posterior pedicle screw fixation

Statistically significant at p < 0.05

### Complications

There were no injuries to blood vessels, nerves, sympathetic nerve chains, or abdominal organs or other serious complications in the two groups. There were cases of endplate injury in the OLIF-SA and OLIF-PPS groups, but no further progress was made after the operation or during follow-up. In the OLIF-SA group, there were 53 patients with cage subsidence after the operation; 52.94% (45/85) of the patients exhibited Grade I, 9.41% (8/85) of the patients exhibited Grade II-III, and 2 patients underwent two-stage posterior pedicle screw fixation. In the OLIF-PPS group, only 11.11% (6/54) of the patients had cage subsidence after the operation, and all of them exhibited Grade I. In addition, three patients in the OLIF-PPS group had back myofascial pain, but all of them improved after local conservative treatment, more information displayed in the Table 5.

**Table 5:** Complications

	SA group	PPS group
Endplate damage	4(4.71%)	2(3.70%)
Gastrointestinal reactions	3(3.53%)	1(1.85%)
Cage subsidence		
Grade 0	32(37.65%)	48(88.89%)
Grade I	45(52.94%)	6(11.11%)
Grade II	6(7.06%)	
Grade III	2(2.35%)	
Grade IV		
Myofascial pain	0	3(5.56%)

Values are presented as frequency (percentage)

SA: Oblique lumbar interbody fusion stand alone; PPS: Oblique lumbar interbody fusion with posterior pedicle screw fixation

Statistically significant at p < 0.05

## Discussion

With the progress of navigation systems, robot systems and other auxiliary surgical technologies, OLIF technology has also rapidly developed and become widely used and has achieved satisfactory therapeutic effects in a variety of lumbar degenerative diseases [20–23]. Grade I and II degenerative lumbar spondylolisthesis has become a good indication for OLIF surgery. However, OLIF-SA is believed to be prone to postoperative complications of cage subsidence [8]. It has been reported that the cage subsidence rate of OLIF-SA is approximately 30% [8, 24]. However, not all cases of cage subsidence need to be treated.

We have found that there is a great difference in interbody fusion between patients who undergo OLIF-SA and those who undergo OLIF-PPS, but no report has been published. In addition, to our knowledge, this retrospective study had the largest sample size and the most research variables for evaluating the results of the treatment of degenerative lumbar spondylolisthesis with OLIF-SA and OLIF-PPS.

Similar to previous reports, in our study, the OLIF-SA group had a shorter operation time, less blood loss and a similar hospitalization period compared to the OLIF-PPS group [10, 16]. This is because the patients in the OLIF-PPS group changed positions during the operation from the lateral position to the prone position and had more incisions on their back. The research of Zhong Dai et al. Mentioned that in addition to the dominant blood loss, there are also a large number of hidden blood loss in the interbody fusion surgery. The risk is higher in patients with diabetes, hypertension and other diseases at the same time [25]. Giuseppe Maccagnano et al.‘s study showed that intraoperative and postoperative use of Tranexamic acid can effectively reduce bleeding [26]. In the future, the wide application of the Mazor x stealth edition and other robots supporting lateral screw placement may reduce this difference. The patients in both groups all wore a thoracolumbar brace on the first day after the operation, so there was no significant difference in the length of stay.

We found that both OLIF-SA and OLIF-PPS were able to restore DH through surgery, but the long-term maintenance of DH in the OLIF-SA group was significantly worse than that in the OLIF-PPS group. However, OLIF achieves indirect decompression by opening the intervertebral space and enlarging the height of the intervertebral space and the foraminal area, which means that the surgical efficacy of OLIF-SA may decrease over time; cage subsidence is also an important factor in revision after OLIF surgery [27, 28]. In OLIF-SA, Pivox, the lateral plate, can be used for auxiliary fixation. Zhang, Guo, and He et al. reported that lateral plate fixation was superior to OLIF-SA in avoiding cage subsidence [11, 14, 16], but the study by Ge et al. showed that additional lateral plate fixation was ineffective in preventing subsidence [12]. However, current reports generally suggest that OLIF-PPS can improve the axial load-bearing capacity of the fusion segment, thereby reducing the incidence of subsidence. In addition, we found that the OLIF-PPS group had a significantly better distance of spondylolisthesis reduction than the OLIF-SA group. This may be due to the pulling reduction effect of the screw rod during posterior pedicle screw fixation, which also explains the increased improvement in the FSA and LA of the OLIF-PPS group.

**Fig. 4:**
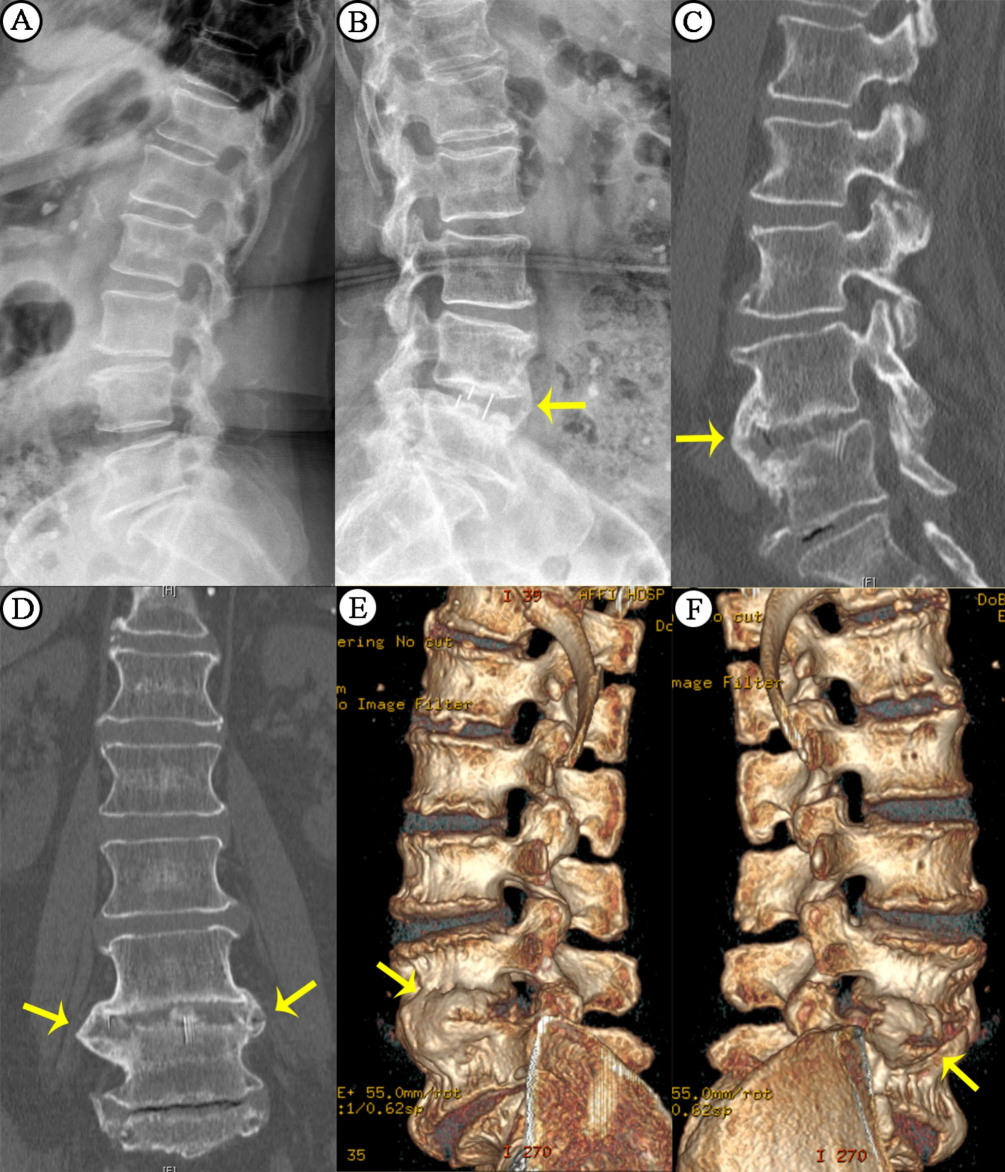
A 71-year-old woman in the SA group underwent OLIF stand-alone. Preoperative x-rays showed no osteophyte formation in the intervertebral space or vertebral edge (**A**). The yellow arrow on the postoperative X-ray and computed tomography scans showed that osteophyte formats at the edge of vertebral body (**B**-**D**). The yellow arrow on computed tomography scans three-dimensional imaging showed osteophyte formation on the left and right sides of the anterior edge of the vertebral body (**E**-**F**).

In our study, both groups achieved a 100% fusion rate at 12 months post-operatively, while the fusion rate in the OLIF-SA group was higher than that in the OLIF-PPS group at 6 months post-operatively, but the difference was not significant. In addition, the proportion of AO-type fusion in the OLIF-SA group was significantly higher than that in the OLIF-PPS group. We believe that the postoperative stability of the OLIF-PPS fusion segment is superior to that of the OLIF-SA group. The fixation of pedicle screws stabilizes the lumbar three-column structure. However, the OLIF-SA fusion segment exhibits slight motion, which means that there is relative displacement between the cage and the upper and lower endplates that does not affect structural stability; this effectively stimulates osteophyte formation around the cage that connects, forming peripheral fusion. Considering the size of the cage, BO-type fusion implies a larger fusion area, which may result in a more robust fusion structure (Fig. 4).

In our study, the VAS score and ODI of both groups gradually improved at various stages of postoperative follow-up. At only one week of follow-up, the VAS score and ODI of back pain in the OLIF-SA group were lower than those in the OLIF-PPS group due to the additional back incisions and the insertion of pedicle screws in the OLIF-PPS group. This also demonstrated the advantage of OLIF-SA in protecting posterior paravertebral soft tissue. This difference disappeared at subsequent follow-up visits.

In addition to cage subsidence, OLIF has the risk of damaging important structures, such as the abdominal great vessels, lumbar plexus, abdominal organs, and ureters, due to its unique approach to the retroperitoneal psoas major muscle space [9, 29, 30]. However, in our study, there were no such serious complications; only a few postoperative complications with mild symptoms occurred, and they improved after observation or conservative management.

This study had some limitations. First, there was a slight difference in the number of patients between the two groups, which may affect the differences in results. Second, all the patients were from the same medical center, which may lead to some limitations and biases. In addition, speculation about the fusion type was based only on the results of imaging examinations, and we hope that further studies from multiple centers will be performed.

## Conclusion

For mild degenerative lumbar spondylolisthesis, we believe that OLIF-PPS can achieve a better reduction effect and long-term maintenance of efficacy. However, compared to OLIF-SA, this technique may have defects such as a longer operative time and damage to the paravertebral muscles. In addition, we found that the fixation method of OLIF-SA leads to the fusion of the surgical segment starting at the edges, which we called the AO type. However, OLIF-PPS tends to lead to central fusion, which we called the BO type. The AO type has a larger fusion area. For different populations, we believe that OLIF-SA or OLIF-PPS are excellent surgical methods.

## Data Availability

The data used and analyzed during the current study are available from the corresponding author upon reasonable request.
